# Validation of the CRAVE-C scale in Chinese adults: a four-study examination of competing motivations for physical activity versus rest

**DOI:** 10.3389/fpsyg.2024.1467949

**Published:** 2024-10-23

**Authors:** Zhihui Cheng, Alyx Taylor, Matthew A. Stults-Kolehmainen, Markus Gerber, Fabian Herold, Michael Ross, Garrett Ash, Arthur F. Kramer, Mengxian Zhao

**Affiliations:** ^1^School of Physical Education, Shenzhen University, Shenzhen, China; ^2^School of Health and Rehabilitation Sciences, AECC University College, Bournemouth, United Kingdom; ^3^Yale New Haven Hospital, New Haven, CT, United States; ^4^Teachers College, Columbia University, New York, NY, United States; ^5^Department of Sport, Exercise and Health, University of Basel, Basel, Switzerland; ^6^Research Group Degenerative and Chronic Diseases, Movement, Faculty of Health Sciences Brandenburg, University of Potsdam, Potsdam, Germany; ^7^Physical Activity Laboratory, Baker Heart & Diabetes Institute, Melbourne, VIC, Australia; ^8^Institute for Physical Activity and Nutrition (IPAN), School of Exercise and Nutrition Sciences, Deakin University, Geelong, VIC, Australia; ^9^VA Connecticut Healthcare System, West Haven, CT, United States; ^10^Beckman Institute for Advanced Science and Technology, University of Illinois at Urbana-Champaign, Champaign, IL, United States; ^11^Center for Cognitive and Brain Health, Northeastern University, Boston, MA, United States; ^12^Beckman Institute, University of Illinois Urbana-Champaign, Champaign, IL, United States

**Keywords:** motivation, physical activity, psychometric validation, sedentary behavior, desire

## Abstract

**Background:**

The study aimed to validate the Cravings for Rest and Volitional Energy Expenditure (CRAVE) scale among Chinese adults with different health conditions (healthy control, chronic illnesses, and psychiatric disorders) and skill levels (athletes vs. non-athletes).

**Methods:**

In Study 1, a confirmatory factor analysis (CFA) using the Maximum Likelihood Method (MLM) was performed on a Chinese sample of emerging adults (*N* = 481) to evaluate the structural validity of the Cravings for Rest and Volitional Energy Expenditure-Chinese version (CRAVE-C). In Study 2, differences in “Move” and “Rest” desires were examined among patients with psychiatric disorders, patients with chronic illnesses, and healthy controls. In study 3, investigated the relationship between cardiorespiratory fitness and exercise motivation using the CRAVE-C (*N* = 83). In Study 4, the changes in “Move” desire from baseline to post-training were compared between athletes and non-athletes.

**Results:**

Results from Study 1 indicated that the 10-item CRAVE-C showed good fit indices ( *Chi^2^* (34) = 118.769, *CFI* = 0.95, *TLI* = 0.934, *SRMR* = 0.053, *RMSEA* = 0.072). “Move” positively correlated with various factors of the Affective Exercise Experiences Questionnaire-Chinese and the Physical Effort Scale-Chinese, while “Rest” correlated negatively. In Study 2, Patients with psychiatric disorders had a significantly higher “Move” desire than healthy controls. Patients with chronic illnesses had a significantly higher “Rest” desire than healthy controls. In Study 3, higher cardiorespiratory fitness was associated with a slight increase in “Move” desire (3.26% ± 37.35%) and a decrease in “Rest” desire (18.94% ± 66.99%). Lower fitness was linked to a significant decline in “Move” desire (−54.61% ± 111.33%) and an increase in “Rest” desire (43.62% ± 63.64%). In Study 4, the athlete group demonstrated a significant increase in “Move” desire from baseline to post-training, whereas the non-athlete group reported a significant decrease in “Move” desire from baseline to post-training.

**Conclusion:**

The 10-item CRAVE-C has good reliability and validity in the Chinese cultural context and can be used among Chinese adults with different health conditions and skill levels.

## Introduction

Inadequate physical activity and sedentary behavior (SB) pose a growing challenge to the global healthcare system, as both of these unhealthy lifestyle behaviors are associated with detrimental health effects (Chen et al., 2021; Cristi-Montero et al., 2019; Ekelund et al., 2024), including but not limited to obesity (Barnett et al., 2018), cardiovascular disease (Lavie et al., 2019), diabetes (Piercy et al., 2018), neurological (Maasakkers et al., 2022; Zou et al., 2024) and psychiatric disorders (Ba et al., 2024; Huang et al., 2020; Werneck et al., 2023), and premature death (Warburton et al., 2006). Specifically, sedentary behavior has been shown to negatively impact cardiovascular health by altering hemodynamics, reducing blood flow, and impairing vascular endothelial function, leading to increased arterial stiffness and elevated cardiovascular disease risk (Carter et al., 2017). Additionally, prolonged sedentary time is linked to insulin resistance and metabolic dysfunction, which disrupts glucose regulation and significantly raises the risk of developing type 2 diabetes (Wilmot et al., 2012). To counteract the negative health effects of inadequate physical activity and SB, the World Health Organization (WHO) recommends that adults should engage in at least 150 min of moderate-intensity physical activity (PA) or 75 min of vigorous-intensity PA per week and reduce their time spent in SB (Bull et al., 2020). Accumulating evidence indicates that regular engagement in PA is positively linked to better mental and physical health outcomes (Anderson and Durstine, 2019; Cillekens et al., 2020; Hou et al., 2024; Posadzki et al., 2020; Zhang et al., 2022; Zou et al., 2023). However, a considerable proportion of adults do not meet the amount of PA recommended by the WHO (Bull et al., 2020), as evidenced by population-based surveys conducted in 2016 indicating that more than a quarter of 1.9 million adults were insufficiently physically active (Guthold et al., 2018).

Given this gap between recommended and achieved levels of PA, it is important to better understand what drives or hinders individuals to engage in PA, so that we can better inform future public health interventions. Considering that people tend to be sedentary in most situations (Caldwell et al., 2023; Stults-Kolehmainen, 2023), studies have started investigating the motivational processes of PA (Epstein et al., 2011; Ha and Ng, 2015). Historically, research into the motivational processes of PA has mostly focused on cognitive, reflective processes (Stults-Kolehmainen et al., 2020), but in recent years there has been a noticeable shift towards affective processes (Cheval and Boisgontier, 2021; Williams and Bohlen, 2019; Williams and Evans, 2014; Zenko and Ekkekakis, 2019). Traditionally, motivation related to PA (including structured forms such as physical exercise) and SB has been viewed as a relatively stable trait, primarily within the framework of self-determination theory (Frijda, 2010; Teixeira et al., 2012; Wilson et al., 2008; Zenko and Ekkekakis, 2019). However, recent developments underscore the pivotal role of motivational states that are more variable and subject to change, such as desires and urges, in initiating physically active and inactive behavior (Brand and Ekkekakis, 2018; Williams and Evans, 2014), thus considering motivated behavior as a dynamic state with moment-to-moment fluctuations (Frijda, 2010; Frijda et al., 2014). Traditionally, motivational frameworks emphasized cognitive and rational decision-making, suggesting that as long as individuals have enough information (e.g., health benefits and goal relevance), they will overcome the challenges of exercise and decide to change their behavior. However, recent evidence (Brand and Ekkekakis, 2018; Williams and Rhodes, 2023) has shown that affective responses during exercise, particularly changes in core affective valence, play a significant role in determining future exercise behavior. For instance, Ekkekakis (2017) found that when exercise intensity approaches the lactate threshold, many individuals experience a decrease in pleasure or even discomfort, which is directly associated with future exercise behavior. Similarly, Williams (Williams and Rhodes, 2023) demonstrated that positive affective states during exercise are better predictors of future exercise than purely rational cognition. This shift in the research framework underscores the importance of affective responses, highlighting that individuals are more likely to pursue physical activity for the positive emotional experiences rather than relying solely on rational cognition.

Despite advances in the concept, the development of measurement tools to assess affectively-charged motivational states has lagged behind tools for other motivational constructs (Brand and Ekkekakis, 2018; Williams and Rhodes, 2023). Therefore, the development of the Cravings for Rest and Volitional Energy Expenditure (CRAVE) scale represents an important step forward in quantifying affectively charged motivational states (Stults-Kolehmainen et al., 2021). The CRAVE scale, which initially consisted of 13 items, assesses desires/wants for both PA (i.e., being active) and SB (i.e., resting). It has 10 items that contribute to the scoring (5 items for the “Move” and 5 items for the “Rest” subscales). In a series of five validation studies, Filgueiras et al. (2023) and Stults-Kolehmainen et al. (2021), confirmed that the 10-item CRAVE has good psychometric properties. Specifically, when tested in an American population of 444 young adults, the CRAVE scale demonstrated high reliability (McDonald’s *ω* for “Move” and “Rest” = 0.97) and good test–retest reliability, with interclass correlations over one laboratory session for “Rest” (ICC = 0.69–0.88) and “Move” (ICC = 0.72–0.95) exceeding those observed over 2 years (“Rest”: ICC = 0.49; “Move”: ICC = 0.53), suggesting that the scale measures states rather than traits. Additionally, substantial changes were observed in response to a maximal, graded treadmill test, with decreased “Move” desire (Cohen’s *d* = 1.05) and increased “Rest” desire (Cohen’s *d* = 0.82). The CRAVE scale showed associations with psychosomatic sensations, such as energy, fatigue, tiredness, and deactivation (Stults-Kolehmainen et al., 2021). Notably, the CRAVE scale was also recently validated in 1168 Brazilian participants (mean age = 30.6; 71.6% female) (Filgueiras et al., 2023). Exploratory factor analyses identified two inversely related oblique factors (“Move” and “Rest”) with excellent fit (GFI = 1.00, RMS*R =* 0.03) and high reliability (Cronbach’s *α*: 0.93 and 0.92 respectively) (Filgueiras et al., 2023).

In study1, the primary aim was to validate the CRAVE-C in a Chinese cultural context. We compared the truncated 10-item model with the 13-item model, as used in Western populations, to determine its applicability in China. Additionally, the relationships between the “Move” and “Rest” desires within the CRAVE-C were examined. To establish convergent validity, the CRAVE-C was assessed alongside the Affective Exercise Experiences Questionnaire-Chinese (AFFEXX-C) and the Physical Effort Scale-Chinese (PES-C), focusing on how these scales relate to motivational states such as approach and withdrawal forces.

In Study 2, the aim was to investigate differences in the CRAVE-C dimensions of “Move” and “Rest” between individuals with psychiatric disorders, chronic illnesses, and a healthy control group. Accumulating evidence suggests that those with conditions like schizophrenia and type 2 diabetes engage in lower levels of PA and higher levels of SB, which are linked to disease progression and mortality (de Wit et al., 2011; Sanchez-Villegas et al., 2008; Schuch et al., 2017; Vancampfort et al., 2015). Therefore, understanding the psychological factors underlying these behaviors in different populations is crucial. By exploring the differences in “Move” and “Rest” motivations across these groups, this study can identify unique psychological drivers that may contribute to their health behaviors. This insight is essential for developing targeted public health strategies aimed at promoting PA and reducing SB, ultimately improving health outcomes in these populations.

In Study 3, the relationship between cardiorespiratory fitness, as measured by VO2 max, and self-reported “Movement desires” (“Move” and “Rest”) was explored under stringent laboratory conditions (Matthews et al., 2016). While VO2 max is widely recognized as a marker of aerobic fitness (Berthouze et al., 1995; Giacobbi et al., 2006; Martin-Rincon and Calbet, 2020; Schembre and Riebe, 2011), limited research exists on its association with real-time fluctuations in the desire to be physically active (Filgueiras et al., 2023; Taylor et al., 2022). This study represents the first attempt to link the self-reported CRAVE scale with objective physiological markers, such as blood lactate concentrations (Taylor et al., 2022). By exploring this novel connection, the study aims to provide a more comprehensive understanding of the physiological underpinnings that contribute to differences in the “Move” and “Rest” desires across various populations. This insight could help explain why certain individuals exhibit varying motivations for physical activity and sedentary behavior.

In Study 4, based on findings from Study 3, it was observed that individuals with higher cardiorespiratory fitness showed a slight increase in “Move” desires and a modest decrease in “Rest” desires. This study focused on comparing athletes and non-athletes in their responses to high-intensity interval training (HIIT). The hypothesis was that athletes, due to their superior cardiorespiratory fitness from continuous training, would exhibit a greater increase in PA-related desires and a more significant decrease in SB-related desires following the HIIT session.

## Study 1

### Materials and methods

#### Participants

In April 2023, 635 individuals voluntarily participated in Study 1, in which they were asked to complete an electronic questionnaire via the “Questionnaire Star” online platform. Before starting the questionnaire, detailed information was provided to participants regarding the study’s purpose, procedures, potential risks, benefits, and their rights, including the right to withdraw at any time without penalty. Participants were recruited through public invitations shared by Body–Brain-Mind (BBM) Laboratory members on social media platforms, and all participants were healthy undergraduate and graduate students currently enrolled at their respective institutions. This informed consent process was clearly displayed at the beginning of the questionnaire to ensure participants were fully aware and consented before proceeding. Data from participants who completed the questionnaire in less than the required 15 min, as determined by a preliminary test conducted by 12 BBM members, were excluded to maintain response reliability, resulting in 481 valid responses for analysis.

To safeguard participant privacy, all personal identifiers were removed from the dataset before analysis, ensuring anonymity and confidentiality. No financial incentives were offered; participants were primarily motivated by their interest in the subject matter and a desire to contribute to scientific knowledge. This intrinsic motivation ensured high engagement and commitment to the study. The ethics of the study were upheld to the highest standards, with approval from the Ethics Committee of Shenzhen University (NO: PN-2022-00075).

#### Translation procedures

Prior to translating the CRAVE into the Chinese language, an investigator from the BBM Laboratory contacted the corresponding author of the original study (MSK) to obtain permission. Once permission was granted, the BBM team began the translation process. Similar to our previous validation studies (Kuang et al., 2023; Kuang et al., 2022; Wang et al., 2023), the translation process in Study 1 strictly adhered to the recommended procedures for the cross-cultural adaptation of self-report questionnaires (Beaton et al., 2000). A Zoom meeting was conducted in which the original author was invited to discuss and ensure that the translation accurately retained the original meanings. This translated version was subsequently reviewed by an expert panel, consisting of five sport psychologists, two health psychologists, and one psychological assessment specialist, to gather comprehensive feedback. Incorporating their insights, a refined version was then distributed to an initial sample of 11 Chinese college students, majoring in sports-related subjects, to test its readability and clarity. A professor fluent in both English and Chinese, but unfamiliar with study aim, undertook a back-translation. Finally, this version was then used to compare with the original scale to ensure semantic accuracy, which in turn generated the Chinese version of the CRAVE scale (CRAVE-C).

#### Procedure

In April 2023, 635 individuals voluntarily participated in Study 1 in which they were asked to complete an electronic questionnaire via the “Questionnaire Star” online platform. Notably, a test conducted by 12 BBM members indicated that a minimum of 15 min is needed to complete all questionnaires. For those participants who spent time below the threshold, they were excluded, leading to 481 valid responses that were retained for data analyses. Demographic information is presented in Table 1. No participants received financial incentives, and the study was approved by the Ethics Committee of the Shenzhen University (NO: PN-2022-00075).

**Table 1:** Demographics, BMI, and CRAVE-C, AFFEXX-C, and PES-C (*N* = 481).

Variable	*N*	Percent
Gender		
Male	222	46.2%
Female	259	53.8%
Educational level		
Undergraduate	430	89.4%
Master’s degree and above	51	10.6%
Marital status		
Single	300	60.23%
In a non-marital relationship	149	30.9%
Married	30	6.2%
Divorced	1	0.2%
Widowed	1	0.2%

	*M*	*SD*	Shapiro–Wilk *W*	Shapiro–Wilk *p*
BMI	21.68	3.21	0.918	<0.001
Move	33.83	9.24	0.976	<0.001
Rest	23.94	10.71	0.988	0.003
Interest vs. Boredom	−5.28	6.66	0.938	<0.001
Showing off vs. Shying away	−5.61	3.09	0.977	<0.001
Liking vs. Disliking exercise in groups	−9.64	4.87	0.949	<0.001
Competence vs. Incompetence	21.41	4.87	0.942	<0.001
Energy vs. Tiredness	14.72	4.37	0.958	<0.001
Calmness vs. Tension	−7.92	3.82	0.941	<0.001
Pleasure vs. Displeasure	16.34	3.88	0.915	<0.001
Attraction vs. Antipathy	8.77	6.43	0.971	<0.001
Core affective exercise experiences	23.25	9.3	0.962	<0.001
Approach score	13.15	3.59	0.973	<0.001
Avoid score	11.28	3.84	0.975	<0.001

BMI, Body Mass Index; “Move” and “Rest” are subscales from the CRAVE-C; the AFFEXX-C consist of constructs: (i) Interest vs. Boredom; (ii) Showing off vs. Shying away; (iii) Liking vs. Disliking exercise in groups, Competence vs. Incompetence, Energy vs. Tiredness, Calmness vs. Tension, Pleasure vs. Displeasure; (iii) Attraction vs. Antipathy; Approach and Avoidance scores are subscales of the PES-C.

#### Measures and demographic information

The survey included demographic information and three self-reported scales (i.e., CRAVE-C, AF-FEXX-C, and PES-C). In addition, demographic variables were collected, including gender, age, marital status (i.e., single, unmarried, and married), and educational level (i.e., bachelor’s degree, master’s degree and higher).

#### Cravings for rest and volitional energy expenditure-Chinese version (CRAVE-C)

The CRAVE-C was used to measure motivational states for PA and SB, and it contains 13 items that are divided into two dimensions (“Move” and “Rest”) (Stults-Kolehmainen et al., 2021). Notably, only 10 items were used to calculate scores for “Move” desire and “Res” desire, with each dimension including 5 items, while three filler items are considered as alternatives. For example, items of “Move” dimension are: (i) “I want to move my body”; (ii) “I want to expend some energy.” Examples items for “Rest” are: (i)"I want to do nothing active”; (ii)"I want to be motionless.” An example of a filler item is: (i)"I want to burn some calories.” Each item was rated on an 11-point Likert-type scale ranging from 0 (not at all) to 10 (more than ever). Internal consistency for the Move factor was tested with Cronbach’s *α* coefficients, yielding a value of 0.89, while the Rest factor demonstrated a Cronbach’s α value of 0.91, both indicating adequate internal consistency.

#### Affective exercise experiences questionnaire-Chinese version (AFFEXX-C)

The AFFEXX-C is an instrument specifically designed to measure affective exercise experiences and it consists of 30 items. There are 8 factors within 3 constructs – namely antecedent appraisals (4 factors), core affective exercise experiences (3 factors), and attraction-antipathy (1 factor). Each item is rated on a 7-point Likert scale. Higher scores on each factor indicate a more positive level of these three constructs (related to better antecedent appraisals [Interest vs. Boredom, Showing off vs. Shying away, Liking vs. Disliking exercise in groups, Competence vs. Incompetence] and core affective exercise experiences [Energy vs. Tiredness, Calmness vs. Tension, Pleasure vs. Displeasure], and more attraction towards physical exercise [Attraction vs. Antipathy]). This instrument had sound psychometric properties among Chinese college students in a previous study carried out by our research group, with all Cronbach’s *α* > 0.8 (Wang et al., 2023). In the present sample (Study 1), the Cronbach’s *α* coefficients for the different subscales ranged between 0.83 and 0.92, indicating good reliability.

#### Physical effort scale-Chinese version (PES-C)

The PES was used to measure tendencies to approach or avoid physical effort and it contains eight items that are divided into two subscales: (i) “Approach” (e.g., “I seek out tasks that require physical effort”); (ii) “Avoidance” (e.g., I avoid tasks that require physical effort whenever possible) (Cheval et al., 2024). Responses to all items are given on a 5-point Likert-type scale from 1 (strongly disagree) to 5 (strongly agree). Validated in a sample of 481 participants, the scale shows adequate internal consistency (“Approach”: Cronbach’s *α* = 0.91, “Avoidance”: Cronbach’s *α* = 0.90), a well-fitting two-factor structure confirming its structural validity [Chi^2^ (19) = 69.243, *p <* 0.001, CFI = 0.959, TLI = 0.939, SRMR = 0.032, RMSEA = 0.074, *p* ≤ 0.08 = 0.016], convergent validity (*β* = 0.50–0.77), as well as discriminant validity (β = 0.10–0.33) (Cheval et al., 2024). In the present sample (Study 1), confirmatory factor analysis (CFA) supported the two-factor structure (Approach and Avoidance) of the PES (Chi^2^ (19) = 69.237, *p <* 0.001, CFI = 0.959, TLI = 0.940, SRMR = 0.032, RMSEA = 0.074), with Cronbach’s *α* coefficient of internal consistency for “Approach” (0.89) and “Avoidance” (0.89) dimensions pointing towards adequate reliability.

#### Statistical analyses

Height and weight were used to compute Body Mass Index (BMI, kg/m^2^). Descriptive statistics were applied to calculate means and standard deviations (SD). Before the main analyses, the Shapiro–Wilk test was employed to assess the normality of the data distribution. In line with previous validation studies (Stults-Kolehmainen et al., 2021), an Exploratory Factor Analysis (EFA) was conducted using SPSS Version 27.0 (Armonk, NY: IBM Corp.). The Optimal Implementation of Parallel Analysis (OIPA) was applied to determine the number of dimensions. Factor extraction was achieved via the Unweighted Least Squares (ULS) approach, followed by oblique rotation that was employed to refine the factors. To ensure the suitability of the correlation matrix, the Bartlett test was utilized, with an anticipated significance of *p <* 0.05 and the Kaiser-Meyer-Olkin (KMO) test, aiming for a result above 0.80. Adhering to Kelley and Lorenzo-Seva’s guidelines (Lorenzo-Seva and Ferrando, 2006), the explained variance of factors and factor loadings of >0.3 were used to determine which dimension (“Move” or “Rest”) each item belongs to. Second, to further confirm the factor structure of the CRAVE-C, confirmatory factor analysis (CFA) with Maximum Likelihood Estimation was conducted based on 481 participants of Study 1, via Mplus (Version 8). To determine model fit, several goodness-of-fit indices were used as suggested by Hu and Bentler (1995) and Kline (2023): (1) Chi-square test (χ^2^) with a significance level of *p >* 0.05 indicating a satisfactory fit, (2) Comparative Fit Index (CFI) with values of 0.90 or above reflecting a good fit, (3) the Tucker-Lewis Index (TLI) with values of 0.90 or higher suggesting adequate fit, (4) the Standardized Root Mean Square Residual (SRMR) with values below 0.08 indicating a proper fit, and (5) the Root Mean Square Error of Approximation (RMSEA) with scores below 0.08 representing good fit. Notably, based on the original 10-item CRAVE, 481 Chinese adults were also used for CFA to generate model fit indices. Such results were used to compare with model fit indices of the two Chinese versions (the 10-item CRAVE and the 13-item CRAVE). Finally, concurrent validity and internal consistency were analyzed in Study 1. Specifically, concurrent validity was tested using the Spearman correlations to explore associations of the CRAVE-C (“Move” desire and “Rest” desire) with other self-reported measures (i.e., AFFEXX-C and PES-C).

### Results

#### Descriptive statistics

In Study 1, the age of the 481 participants ranged from 18 to 30 years with a mean age of 20.92 (SD = 2.81). The average BMI was 21.68 kg/m^2^ (SD = 3.21). Results are presented in Table 1.

#### Exploratory factor analysis (EFA)

Results from the Kaiser-Meyer-Olkin (KMO) was 0.856 while the Bartlett test was significant [3325.535 (*df* = 78; *p* < 0.001)], indicating that data in Study 1 is suitable for EFA (Shrestha, 2021). Based on the Eigenvalue-greater-than-one rule, EFA revealed a two-factor structure: (1) 41.34% of variance was explained by Factor 1 (Eigenvalue = 5.733) and 14.47% of variance was explained by Factor 2 (Eigenvalue = 1.996). Further, a moderate negative correlation (*r* = −0.569) was observed between the two (“Move” and “Rest”) factors. The pattern matrix indicated that Factor 1 comprised items 1, 2, 5, 6, 9, and 13, while Factor 2 included items 3, 4, 7, 8, 10, 11, and 12. As shown in Table 2, all factor loadings for these items were greater than 0.30, supporting their inclusion in their respective factors. In summary, results indicated a valid and reliable two-factor model, with the cumulative variance of 55.81%.

**Table 2:** Descriptive statistics and factor loadings for “Move” and “Rest” (*N* = 481).

Item	Descriptive statistics				Factor loading	
	*M*	*SD*	Skewness	Kurtosis	Move	Rest
Move						
1. Move my body	6.75	2.4	−0.82	0.41	0.859	0.039
2. Be physical active	6.56	2.39	−0.70	0.14	0.902	0.028
5. Burn some calories	6.44	2.5	−0.46	−0.33	0.705	−0.032
6. Expend some energy	7.15	2.16	−0.71	0.4	0.801	0.171
9. Exert my muscles	6.42	2.36	−0.56	0.01	0.478	−0.197
13. Move around	6.95	2.24	−0.73	0.41	0.595	0.255
Rest						
3. Do nothing active	4.08	2.63	0.16	−0.79	−0.329	0.343
4. Just sit down	3.86	2.68	0.35	−0.57	−0.411	0.388
7. Be still	6.32	2.34	−0.27	−0.3	0.147	0.333
8. Be a couch potato	4.77	2.74	0.11	−0.66	0.089	0.716
10. Be motionless	4.23	2.7	0.27	−0.6	−0.25	0.57
11. Lay down	4.71	2.76	0.18	−0.69	−0.036	0.834
12. Rest my body	5.91	2.46	−0.38	−0.09	0.197	0.815

The pattern matrix indicated that Factor 1 (“Move”) comprised items 1, 2, 5, 6, 9, and 13, while Factor 2 (“Rest”) included items 3, 4, 7, 8, 10, 11, and 12. All factor loadings for these items exceeded the commonly accepted absolute threshold of 0.30, thereby justifying their inclusion in their respective factors. To conclude, the analysis identified a robust and reliable two-factor structure, which together explains 55.81% of the cumulative variance, highlighting the clear distinction between two main underlying constructs within the dataset.

#### Confirmatory factor analyses (CFA)

As indicated by results in Table 3, the 10-item CRAVE-C outperformed the original 10-item CRAVE and the 13-item CRAVE-C. Specifically, among the three above-mentioned models, the 10-item CRAVE-C demonstrated the best model-fit indices, with the lowest values for AIC (19845.631), BIC (19975.148), RMSEA (0.072), and SRMR (0.053), and with the highest values of CFI (0.950) and TLI (0.934). Notably, the 10-item CRAVE-C had the lowest Chi^2^ value (Chi^2^ (34) = 118.769, *p* < 0.001), compared to the original 10-item CRAVE (Chi^2^ (34) = 162.089, *p* < 0.001) and the 13-item CRAVE-C (Chi^2^ (64) = 319.818, *p* < 0.001).

**Table 3:** Model fit indices for three versions of the CRAVE.

Fit index	10 items (Original version)	10 items (Chinese version)	13 items (Chinese version)
AIC	20200.62	19806.41	26285.049
BIC	20329.514	19935.862	26352.084
RMSEA	0.089	0.072	0.091
CFI	0.910	0.950	0.88
TLI	0.881	0.934	0.854
SRMR	0.063	0.053	0.078

In Study 1, data from 481 participants were utilized to compare three different versions of the CRAVE. The original CRAVE comprised a total of 10 items, selected based on a previous study by Stults-Kolehmainen et al. (2020), with 5 items representing the “Move” subscale (Items 1, 2, 6, 9, 13) and 5 items representing the “Rest” subscale (Items 3, 4, 7, 8, 10). For the newly generated 10-item CRAVE-C among 481 Chinese adults, the selection of items was strategically based on the highest factor loadings from the initial item pool. Specifically, this version included 5 items for the “Move” (Items 1, 2, 5, 6, 9) and 5 items for the “Rest” (Items 4, 8, 10, 11, 12) subscales, ensuring the statistical robustness of the scale. The 13-item CRAVE-C, also among the same cohort of Chinese adults, comprised 6 items for “Move” (Items 1, 2, 5, 6, 9, 13) and 7 items for “Rest” (Items 3, 4, 7, 8, 10, 11, 12) subscales. The exact wording of each item is presented in Table 2.

#### Correlation of CRAVE-C, AFFEXX-C, and PES-C

**Figure 1:**
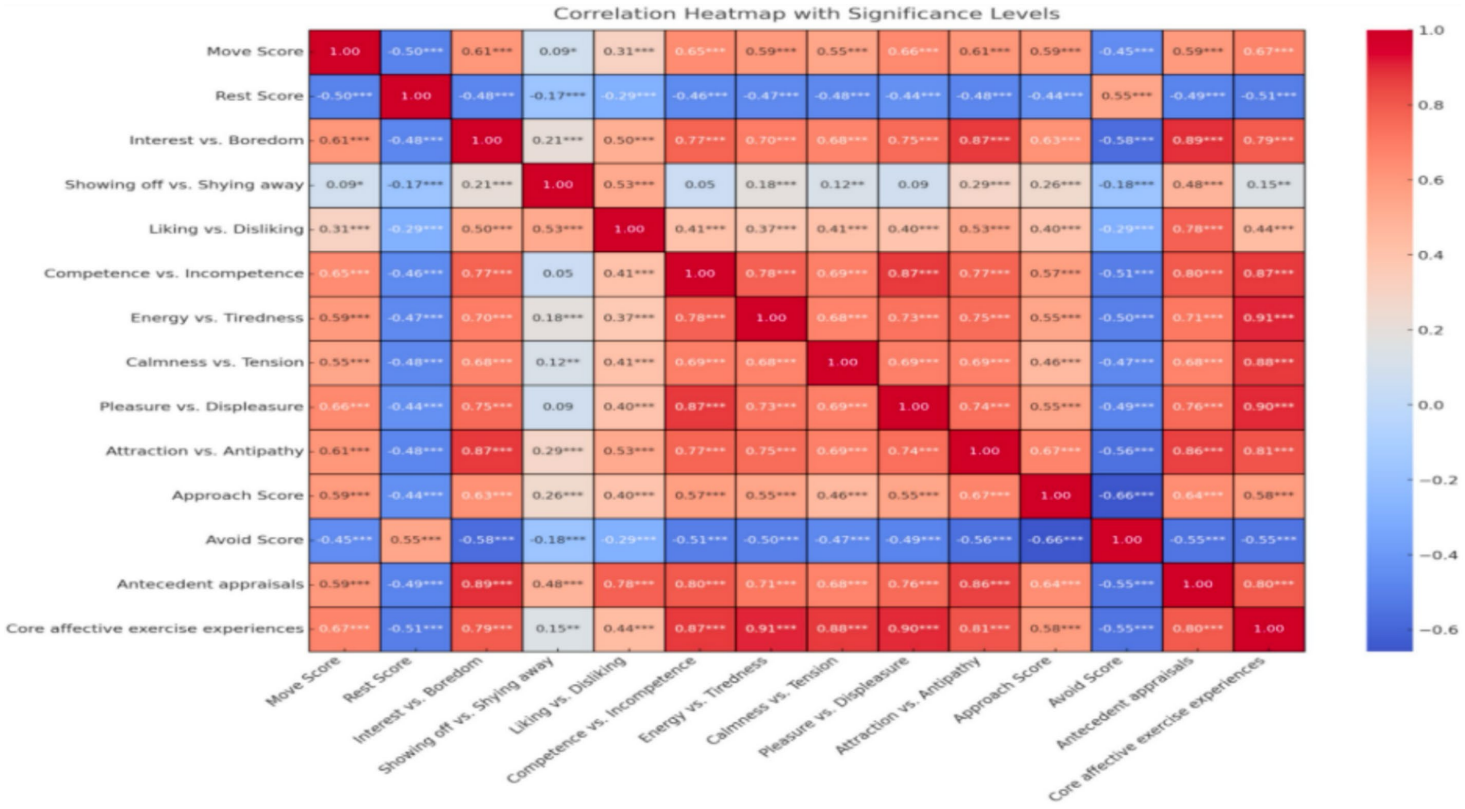
Correlation Heatmap of the 10-item CRAVE-C (*N* = 481). AFFEXX-C consists of antecedent appraisals (Interest vs. Boredom; Showing off vs. Shying away; Liking vs. Disliking exercise in groups, and Competence vs. Incompetence) and core affective exercise experiences (Energy vs. Tiredness, Calmness vs. Tension, and Pleasure vs. Displeasure), and attraction toward physical exercise (Attraction vs. Antipathy); PES-C consists of two Approach and Avoidance; *p < 0.05, **p < 0.01, ***p < 0.001.

“Move” desire was positively correlated with “Pleasure vs. Displeasure “(*r* = 0.66), “Core affective exercise experiences” (*r* = 0.67), “Antecedent appraisals” (*r* = 0.59), and “Attraction vs. Antipathy”(*r* = 0.67) of the AFFEXX-C, whereas all these variables correlated negatively with “Rest” desire (see Figure 1). The “Approach” dimension of the PES-C was positively associated with “Move” desire (*r* = 0.59), and negatively associated with “Rest” desire (*r* = −0.45). The “Avoidance” dimension of the PES-C correlated negatively with “Move” desire (*r* = −0.44) and positively with “Rest” desire (*r* = 0.55). All of the above-presented correlations were significant at *p* < 0.05 level. Detailed results can be found in Figure 1.

### Discussion

To better understand the motivation for PA and SB and to make the CRAVE applicable in research with Chinese-speaking populations, the original instrument was translated into Chinese (CRAVE-C), and its validity and internal consistency were tested among Chinese adults. Results indicate that the CRAVE-C appears to have good psychometric properties and concurrent validity.

Results from our factor analyses were consistent with those from previous studies conducted in the USA (Stults-Kolehmainen et al., 2021) and Brazil (Frijda et al., 2014), supporting a two-factor CRAVE model. Moreover, our results revealed that in the sample of Chinese adults, the 10-item CRAVE-C yielded the best model-fit, but loaded items within “Move” and “Rest” are different from two previous validation studies (Filgueiras et al., 2023; Stults-Kolehmainen et al., 2021). Specifically, the 10 items used in previous studies (Filgueiras et al., 2023; Stults-Kolehmainen et al., 2021) are not all applicable to the 10-item CRAVE-C. For the “Move” subscale, Item 13 was removed and replaced with Item 5. For the “Rest” subscale, Items 3 and 7 were removed and replaced with Items 11 and 12. Such differences in item selection were generated from statistical analyses and 5 items with the highest loading on each factor were finally selected (see Table 1). One reason for these differences may be attributed to the fact that participants were born and grew up within different cultures (Song et al., 2019). For instance, Chinese individuals are less likely to interpret Item 13 (“Move around”) as being in a condition where individuals are engaging in vigorous-intensity PA, instead of being involved with more relaxed PA (e.g., strolling) to help with digestion. Additionally, for “Rest” ‘subscale of the CRAVE-C’, the two items (“Do nothing active,” “Be still”) with the lowest factor loadings (0.343 and 0.333) were removed because Chinese people may be more likely to interpret these two items having the shared elements of “move” desire and “rest” desire. In addition, we examined the separate associations of the CRAVE-C (“Move” desire and “Rest” desire) with the AFFEXX-C and the PES-C. Results suggest that affective exercise experiences may predict an individual’s motivation for PA engagement. Specifically, core affective exercise experiences as one of the key factors of the AFFEXX-C was highly associated with the total scores of “Move” subscale, with *r* = 0.68. This finding implies that Chinese adults who reported a greater level of positive emotional experiences associated with PA are likely to maintain a higher desire for PA engagement. In other words, individuals who enjoy PA tend to have a greater level of “Move” desire, which may in turn be associated with a greater possibility of engaging in PA (de Sousa et al., 2022).

This is the first study to validate the CRAVE in Chinese adults and results from the appropriate statistical analyses indicated the uniqueness of Chinese culture. Second, we built links between the 10-item CRAVE-C and the other two newly developed scales (i.e., AFFEXX-C and PES-C), which help exercise psychologists further substantiate their associations with movement behaviors (e.g., PA and SB) that are not collected in Study 1.

Several limitations of Study 1 need to be acknowledged. First, the use of self-report methods may be subject to social desirability bias, potentially affecting participants’ responses to specific PA-related items in the questionnaire. Second, the study sample predominantly comprised university students in China, lacking representation across a broader age range. Third, the associations between personality and motivation have been well-documented, but the former profile, that has not been collected in the present study, may contribute to their desire for PA engagement. Fourth, fatigue, recent PA, and executive function play a critical role in PA engagement, but these variables were not measured, which may potentially result in the increased or decreased tendency for PA engagement (approach and withdrawal motivational forces). Collectively, the above-presented limitations should be considered for future studies.

## Study2

### Materials and methods

#### Participants

Ninety-five patients were recruited from two different hospitals, comprising 33 adults with psychiatric disorders (including schizophrenia, bipolar disorder, and depression; 18 males, mean age: 33.21 ± 14.41 years, BMI: 23.41 ± 3.77 kg/m^2^) and 62 patients with chronic illnesses (including type 2 diabetes, coronary artery disease, heart failure, asthma, chronic obstructive pulmonary disease, and chronic kidney disease; 32 males, mean age: 45.59 ± 6.13 years, BMI: 24.23 ± 4.21 kg/m^2^). Detailed demographic and clinical characteristics of these patients are provided in Supplementary material 1. All inpatients were diagnosed by clinicians (psychiatrists or physicians). The 481 participants from Study 1 were used as a healthy control group. All participants were fully informed about the nature of the study and provided written informed consent prior to data collection.

#### Statistical analysis

In the sample of 33 adults with psychiatric disorders, Cronbach’s *α* coefficients were 0.88 for “Move” and 0.90 for “Rest.” In the sample of 62 patients with chronic illnesses, Cronbach’s α coefficients were 0.87 for the “Move” and 0.92 for the “Rest” subscales, indicating adequate internal consistency of the CRAVE-C in these two distinct populations. Normality of data distribution was tested using the Shapiro–Wilk test, suggesting mixed results: Data on “Move” (*W* = 0.975, *p* < 0.005) and “Rest” (*W* = 0.99, *p* < 0.005) were not normally distributed in healthy adults, whereas data on patients with psychiatric disorders (“Move,” *W* = 0.936, *p* = 0.052; “Rest,” *W* = 0.958, *p* = 0.226) and adults with chronic illnesses (“Move,” *W* = 0.966, *p* = 0.087; “Rest,” *W* = 0.974, *p* = 0.218) were normally distributed. Given these varied outcomes non-parametric methods were employed. Levene’s test was applied to examine the homogeneity of variance among three groups. Regarding the “Move” and “Rest” subscales, Levene’s test revealed significant differences in variance among the groups (*F =* 4.044, *p* = 0.018; *F =* 7.557, *p* < 0.001 respectively). Kruskal-Wallis *H* test as non-parametric statistics was used to determine whether significant differences on “Move” and “Rest” desires across these groups existed. The magnitude of these differences was quantified using Cohen’s *d*: *d* ≥ 0.2 = small; *d* ≥ 0.5 = medium; *d* ≥ 0.8 = large (Cohen, 1988).

### Results

Patients with psychiatric disorders (25.39 ± 13.47) showed a significantly higher level of “Move” desire relative to healthy controls (25.39 ± 13.47), [*H*(1) = 11.93, *p <* 0.001, *d* = 0.77]. However, a non-significant difference was observed on this outcome measure between healthy controls and patients with chronic illnesses, [*H*(1) = 2.74, *p =* 0.098].

**Figure 2:**
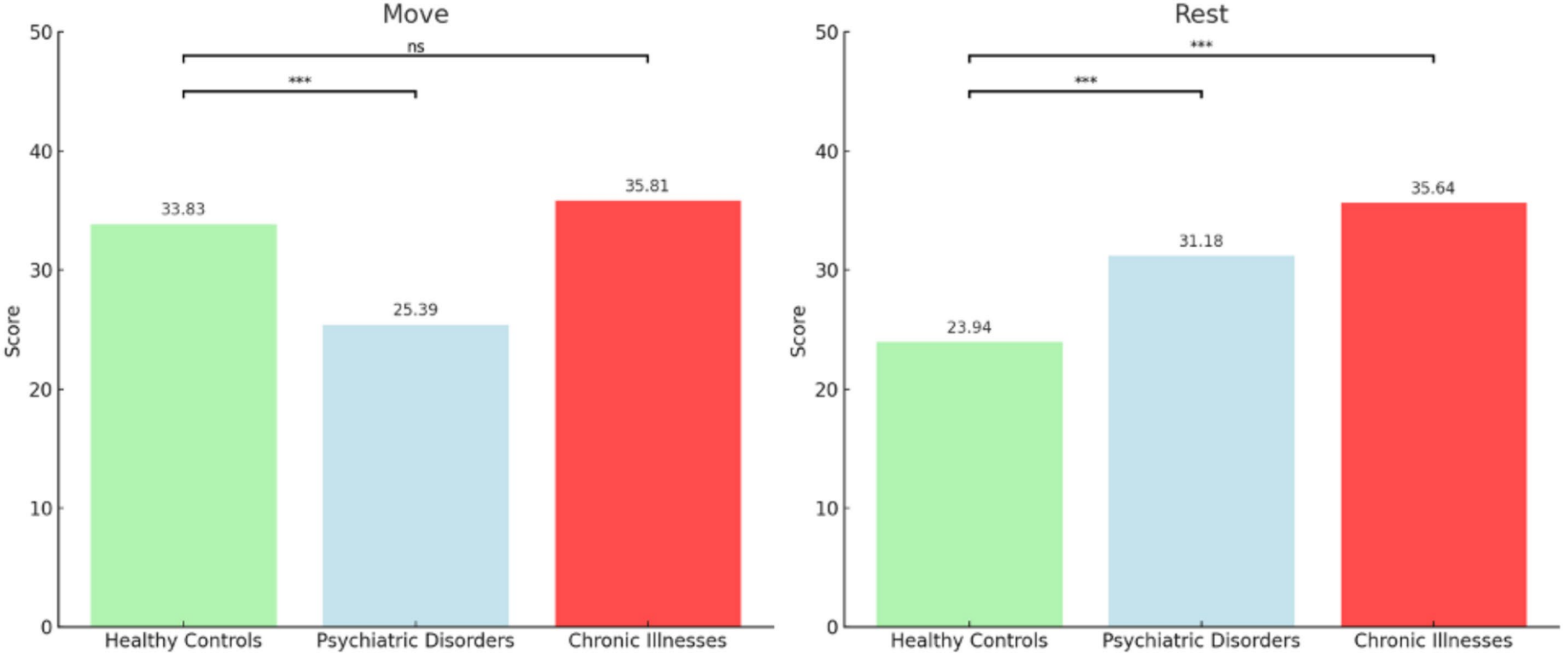
Differences in CRAVE-C “Move” and “Rest” Desires Across Groups. The bars represent mean scores. *p < 0.05, **p < 0.01, ***p < 0.001.

Patients with chronic illnesses showed a significantly higher level of “Rest” desire (31.18 ± 13.04) compared to healthy controls (25.95 ± 9.76), [*H*(1) *=* 10.1, *p* < 0.001, *d* = −0.98]. Patients with psychiatric disorders (35.64 ± 11.22) showed a significantly higher level of “Rest” desire compared to healthy controls (25.95 ± 9.76), [*H*(1) *=* 21.1, *p* < 0.001, *d* = −0.51] (Figure 2).

### Discussion

The study investigated differences in motivational states towards PA (“Move” desire) and SB (“Rest” desire) among healthy individuals, patients with psychiatric disorders, and patients with chronic illness. Results indicated that patients with psychiatric disorders reported the lowest level of “Move” desire. Such findings may be explained by the following reasons. First, in previous research patients with psychiatric disorders demonstrated significantly greater levels of cognitive bias as compared to an age-matched control group (Moritz et al., 2016). The general cognitive biases in this unique group may be closely linked to PA-related cognitive errors, which may in turn result in less PA engagement (REF 57?). Such an assumption has been supported by our recent study, which indicated that patients with psychiatric disorders demonstrated significantly higher levels of exercise-related cognitive errors than healthy controls. (e.g., exaggerating the negative effects of exercise). Interestingly, on the one hand, individuals with chronic illnesses exhibited a greater desire to move as compared to healthy controls. Such a finding may be attributed to the fact that this unique group is aware of the importance of PA engagement for alleviating illness-related symptoms. Especially because in China, PA prescription within the “Healthy Plan 2030” has been heavily promoted in clinical settings (Dai and Menhas, 2020). On the other hand, patients with chronic illness exhibited an increased inclination for rest. Such findings may be attributed to the fact that patients with chronic illnesses often experience physical pain and have a relatively low fatigue threshold that is linked to low exercise tolerance, resulting in an increased desire to rest (White et al., 2010).

The current study has some limitations that should be considered. First, the modest sample size and the limited range of psychiatric and chronic conditions may reduce the generalizability of our findings. Second, the severity of illness was not collected in the present study, which precludes us from making a nuanced interpretation. Despite these limitations, given the scarcity of research exploring the desire for PA and SB in different populations, our study still provides useful information. In future research, we plan to expand our sample size and control for more variables, including severity, type, and length of illness to enhance the accuracy and reliability of our results.

## Study 3

### Materials and methods

#### Participants

A total of 83 participants were recruited for this study. Their ages ranged from 19 to 24 years with a mean age of 21.82 (*SD =* 1.5). The average BMI was 21.74 (SD *=* 3.3). Of these participants, 33 were male and 50 were female.

#### Measures

##### Maximal oxygen uptake (VO₂max) test

The VO₂max test was administered using the COSMED metabolic system through a graded exercise test (GXT) on a bicycle ergometer (DeBlois et al., 2021). The test started at 0 watts, and the workload was increased by 1 watt every 3 s, with a maximum resistance of 250 watts. Participants maintained a cadence of 60–65 rpm throughout the test. This protocol provided a gradual yet consistent increase in exercise intensity, ideal for accurately assessing cardiorespiratory fitness. The test continued until participants either voluntarily stopped due to excessive fatigue or achieved a respiratory exchange ratio greater than 1.1, ensuring that the assessment accurately reflected their maximum cardiorespiratory capacity (Edvardsen et al., 2013). VO₂max values were characterized by the highest recorded oxygen consumption rate (i.e., absolute VO_2_max), adjusted for each individual’s body weight, and reported in units of ml/kg/min (i.e., relative VO_2_max).

##### CRAVE-C (Right now version)

Prior to and following the VO₂max test, participants completed the “Right now” version of the CRAVE-C assessment as described above. Specifically, the questionnaire was administered 1 min before the test commenced and again 1 min after cessation of the test. This tool assesses immediate motivational tendencies towards moving and resting on a scale from 0 (not at all) to 10 (more than ever).

#### Statistical analysis

In this research, the correlations between CRAVE-C subscale scores and VO2max (maximal oxygen uptake) were rigorously explored using SPSS 27.0 for statistical analysis. The CRAVE-C subscale captures key psychological states, specifically “Move” and “Rest,” both before and after exercise interventions. These states were quantified as percentage changes (*Δ*%"Move” and Δ%"Rest”) to assess the impact of physical activity.

Our analysis involved calculating Pearson’s correlation coefficients to determine the relationships between these psychological changes and VO2max. A *p*-value of less than 0.05 was set as the threshold for statistical significance. Participants were stratified into two groups - high and low - based on their VO2max, with the median value of 33.5 mL/kg/min serving as the dividing line. This stratification enabled a detailed comparison of the Δ%"Move” and Δ%"Rest” scores’ mean and standard deviation across the groups. Furthermore, it allowed for an in-depth examination of the correlation of these scores with VO2max within each subgroup.

SPSS 27.0 was used for the analyses. The outcomes of this statistical analysis provide valuable insights into the relationship between exercise-induced mental state alterations and cardiorespiratory fitness, as represented by VO2max.

### Results

The percentage change in “Move” score after exercise (Δ%"Move”) was negatively correlated with the percentage change in “Rest” score (Δ%"Rest”) with a correlation coefficient of *r* = −0.41. Furthermore, Δ%"Move” demonstrated positive associations with the time to complete the CRF test (seconds) with *r* = 0.42, max power output during the GXT (watt) at *r* = 0.41, absolute maximal oxygen uptake (VO₂max) at *r* = 0.48, and the relative maximal oxygen uptake (ml/kg/min) at *r* = 0.56. The percentage change in “Rest” score after exercise (Δ%"Rest”) exhibited negative correlations with absolute VO₂max (*r* = −0.22), and relative VO₂max (*r* = −0.4).

The data reveals a significant contrast: the “Move” desire in the high cardio-pulmonary group increased by 3.26% on average, while it decreased by 54.61% in the low group. Conversely, the “Rest” desire decreased by 18.94% in the high group and increased by 43.62% in the low group. In the high cardiorespiratory group, the correlation of Δ%"Move” with Time and Δ%"Move” with Watt is significantly lower compared to the low cardiorespiratory group. Furthermore, the correlation between Δ%"Rest” and Kg/VO2MAX is completely inverse in the two groups (Table 4).

**Table 4:** Differential cardiorespiratory fitness in High vs. Low Groups.

	High Group	Low Group
Mean and SD of Δ% "Move”	3.26% ± 37.35%	−54.61% ± 111.33%
Mean and SD of Δ% "Rest”	−18.94% ± 66.99%	43.62% ± 63.64%
Δ%"Move” with VO2MAX	0.372	0.44
Δ%"Move” with Kg/VO2MAX	0.579	0.524
Δ%"Move” with Time	0.278	−0.442
Δ%"Move” with Watt	0.246	0.445
Δ%"Rest” with VO2MAX	0.135	−0.003
Δ%"Rest” with Kg/VO2MAX	0.022	−0.22
Δ%"Rest” with Time	0.274	−0.08
Δ%"Rest” with Watt	0.304	−0.08

Groups were classified based on their KG/VO2MAX: those with ≥33.5 mL/kg were categorized as the high cardiorespiratory group, and those with <33.5 mL/kg as the low group. 33.5 mL/kg represents the median value for all participants, and thus, we defined those above this threshold as the high group.

### Discussion

The study provides evidence for associations of cardiorespiratory fitness-related parameters (i.e., absolute VO₂ max and relative VO₂max) with changes (from post-to-pre-exercise) on the CRAVE-C-related scores (referring to desire of “Move” and “Rest”). Specifically, both parameters of CRF were negatively correlated with a more pronounced decline of “Move” desire, whereas both parameters of CRF were negatively correlated with the increase of “Rest” desire. Such results may be attributed to the following reasons. First, a higher absolute and relative VO₂ max, indicative of superior cardiopulmonary health, suggests that adults with higher CRF are capable of tolerating the graded exercise protocol for extended periods of time. This observation aligns with the findings of Marcora and Staiano (2010), suggesting that during high-intensity physical exertion, the critical determinant influencing fatigue perception and exercise sustainment might be the individual’s perceived exertional effort, as opposed to solely muscular fatigue. Consequently, it may be that individuals possessing superior cardiorespiratory capabilities, attributed to habitual engagement in physical training, are likely to demonstrate augmented power outputs and prolonged endurance in response to these modalities of testing, concurrently experiencing a comparatively diminished magnitude of cognitive fatigue (Marcora and Staiano, 2010).

Interestingly, after dividing participants into high and low cardiorespiratory fitness groups based on a median value of 33.5 mL/kg, significant differences in desire scores were observed between the groups. In the higher fitness group, a slight increase of 3.26% in “Move” desire was noted, whereas the corresponding “Rest” desire decreased by 54.61%. This suggests that after undergoing maximal intensity exercise, the high fitness group might have experienced benefits akin to a warm-up, preventing significant fatigue and instead activating their bodies, leading to a decrease in “Rest” desire. In contrast, the lower fitness group showed an 18.94% decrease in “Move” desire and a 43.62% increase in “Rest” desire. Future studies could compare professional athletes and non-athletes (Study 4) to further examine these dissociations. This aligns with the notion that their robust aerobic adaptability facilitates quicker recovery, thus diminishing the immediate necessity for prolonged “Rest.” “Move” motivation might also rebound faster for those with better fitness.(Barak et al., 2011; Knowles et al., 2021; Yaffé, 1983).

Unfortunately, the recovery of desires after the GXT was not tested, and limited data has been generated to explore this issue (Stults-Kolehmainen et al., 2023). Future research should examine how desires to “Move” and be sedentary relate to lactate threshold/ventilatory threshold, in accordance with the Dual Process model (Ekkekakis, 2009). The associations observed with exercise duration and intensity, defined as the maximum resistance achieved, further bolster this understanding. As these metrics increased, indicating heightened effort and capacity during the exercise, there was a more pronounced decline in the desire to “Move.” However, these individuals manifested a lesser inclination to “Rest” post-exercise, possibly alluding to their resilience to exercise-induced fatigue.

Taken together, this study accentuates our knowledge on pivotal roles of maximal exercise and aerobic fitness in determining post-exercise behavioral inclinations. In this study, it was observed that younger adults with higher cardiorespiratory fitness exhibited a less pronounced increase in post-exercise desire for “Rest.” However, it is important to note that these findings are based on correlational analysis, which does not imply causation. Based on these findings, tailored exercise programs can be designed for individuals with varying levels of cardiorespiratory fitness to enhance emotional outcomes. For instance, our results indicate that individuals with higher cardiorespiratory fitness experience an increase in the desire to “Move” after maximal intensity exercise. This suggests that incorporating higher intensity workouts for those with greater aerobic capacity could help maintain elevated desire levels for physical activity throughout the day, thereby enhancing their overall exercise experience.

## Study 4

### Materials and methods

#### Participants

Forty-two participants were recruited for this study. Their overall mean age was 20.02 (SD = 0.95) years. Among them, 22 were certified athletes (20.36 ± 0.95 year; 21.33 ± 1.73 kg/m^2^) with a national second-level and above and they were evenly distributed with 11 males and 11 females. The remaining 20 participants (*M* = 19.35 ± 1.04 year, *SD* = 22.4 ± 2.04 kg/m^2^) were university students with an interest in sports, evenly distributed with 10 males and 10 females.

#### Procedure

##### Measurement of the CRAVE-C

All participants were invited to arrive in the university athletic field. Before commencing any physical activity, all participants were required to fill out the CRAVE-C to capture their baseline score. All participants were asked to perform warm-up exercises, followed by the core training. Afterward, the same CRAVE-C was used to carry out the post-assessment for all participants during the cool-down period.

##### Primary training session

Participants first completed an 8-min jog, serving as a warm-up. This was followed by a 5-min dynamic stretching session. A 50-meter sprint was undertaken as a warm-up stage before the core training started. Given individual difference and training adaptability, both athletes and non-athletes were divided based on gender, including four groups (female athletes, male athletes, female non-athlete, and male non-athlete), in order to successfully complete the core training. HIIT was selected as the core training and each participant was asked to perform five 150-meter sprints with a full-speed and the resting/recovery time of 90 s. During the cool-down, participants were asked to engage in a 10-min musculoskeletal stretching.

#### Statistical analyses

First, the Shapiro–Wilk test was utilized to determine whether data were normally distributed. This step was used to ensure that the data conformed to the assumptions required for further parametric tests. Once normality was confirmed, Levene’s Test was conducted to test the homogeneity of variances between the two groups. The outcome of this test determined the variant of the t-test to be used, either assuming equal or unequal variances. Subsequently, an independent samples t-test was executed to compare the differences in CRAVE-C measurements between the two groups. The significance level was set at *p* = 0.05; any *p*-value below this threshold indicated a statistically significant difference.

### Results

**Figure 3:**
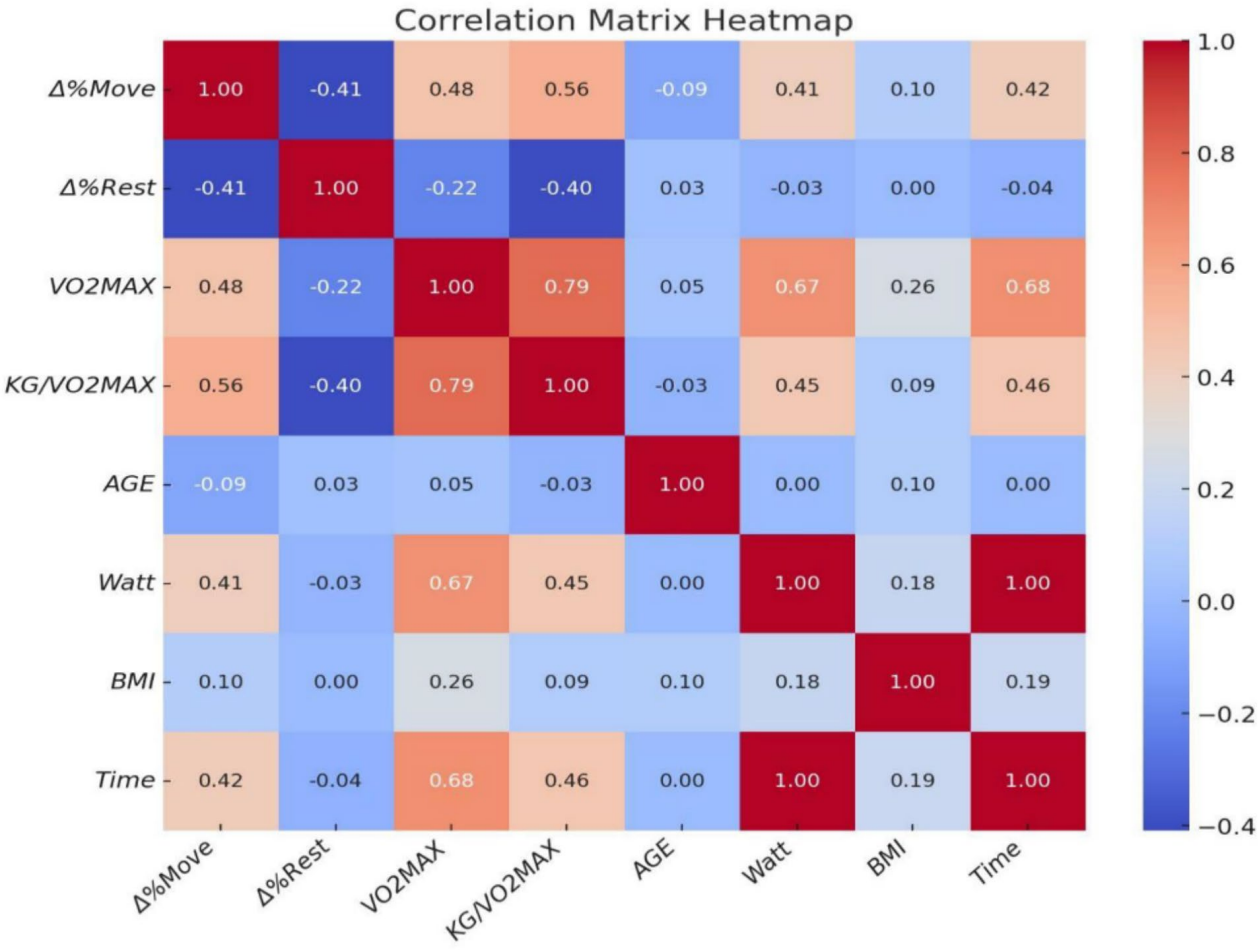
Correlation between pre-and post-test differences and various indicators (*N* = 83). *Δ*% "Move”: Percentage change in “Move” score after exercise; Δ%"Rest”: Percentage change in “Rest” score after exercise; Watt, Max power output during exercise.

**Figure 4:**
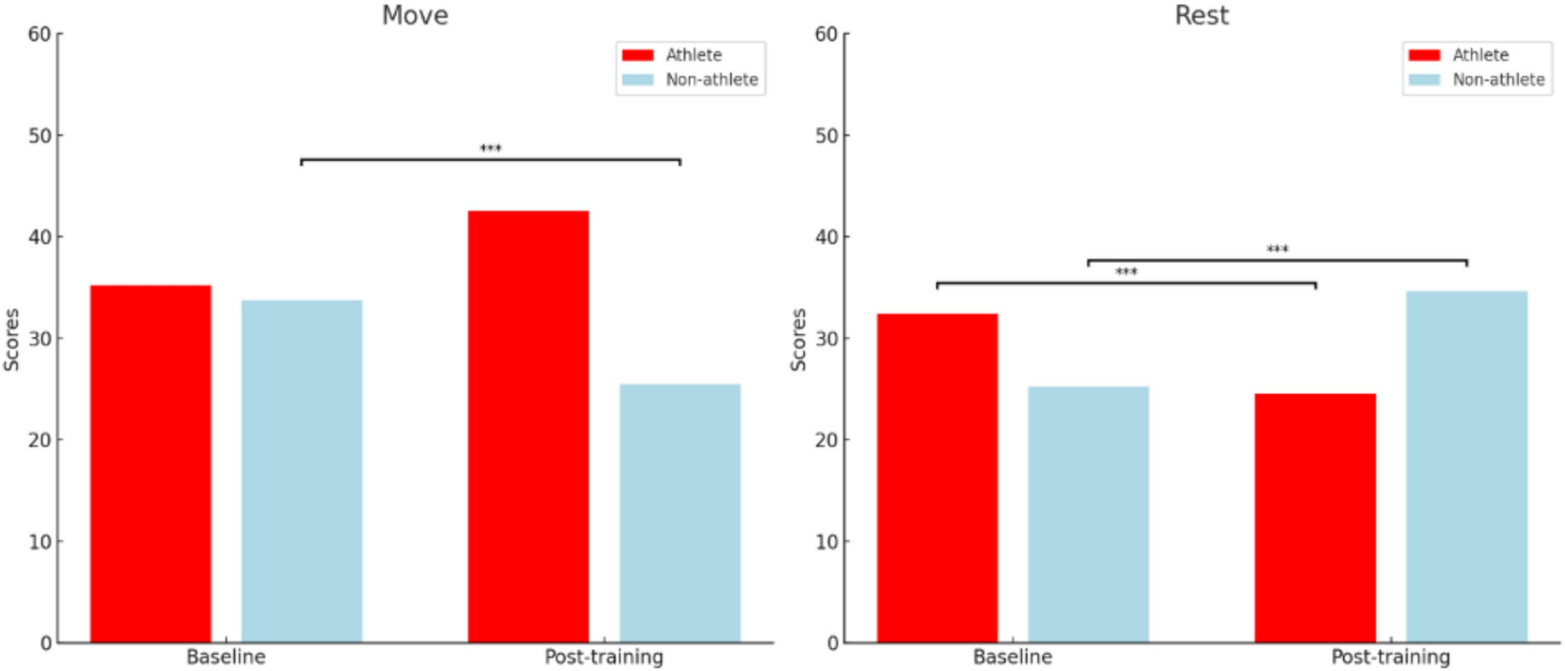
Group difference on the CRAVE-C scores baseline and after training. *p < 0.05, **p < 0.01, ***p < 0.001. The figure compares the scores of athletes and non-athletes in both “Move” and “Rest” categories at baseline and post-training. “Move” refers to the motivation to engage in physical activity, while “Rest” refers to the motivation to rest or reduce physical activity.

For “Move” desire from post-training to baseline, the test yielded *t*(40) = 3.64, *p* < 0.001, indicating that athletes experienced a significantly greater increase in desire as compared to the non-athlete group (Table 3). Notably, the athlete group demonstrated a significant increase in “Move” desire from baseline to post-training, with *t*(21) = 5.2 and *p* < 0.001, whereas the non-athlete group reported a significant “Move” decrease in desire from baseline to post-training [*t*(19) = −3.11, *p* < 0.001]. Conversely, for “Rest” desire from post-to baseline, the test yielded *t*(40) = −7.39, *p* < 0.001, indicating a significant decrease in desire for the athlete group, as compared to the non-athlete group. Notably, the athlete group demonstrated a significant decrease in “Rest” desire from baseline to post-training, with *t*(21) = −3.55 and *p* < 0.001, whereas the non-athlete group reported a significant increase in “Rest” desire from baseline to post-training, with *t*(19) = 6.88, *p* < 0.001. Results for within-group are presented in Figures 3, 4 and Table 5.

**Table 5:** Group difference on the CRAVE-C.

Measure	Athlete (*N =* 22)	Non-athlete (*N =* 20)	Significance
Baseline score on “Move” desire	35.23 ± 7.58	33.75 ± 6.05	*t*(40) = 1.97, *p* = 0.27
*P*ost-training score on “Move” desire	42.56 ± 8.56	25.45 ± 5.01	*t*(40) = 7.86, *p* < 0.001
Significance of Change	*t*(21) = 5.2 *p* < 0.001	*t*(19) = −3.11 *p* < 0.001	
Baseline score on “Rest” desire	32.41 ± 9.19	25.25 ± 6.13	*t*(40) = 3.51, *p* < 0.001
Post-training score on “Rest” desire	24.55 ± 7.20	34.65 ± 8.4	*t*(40) = −2.82, *p* < 0.001
Significance of Change	*t* (21) = −3.55, *p* < 0.001	*t*(19) = 6.88, *p* < 0.001	
“Move”-post-pre-Difference	7.18 ± 12.97	−8.3 ± 7.12	*t*(40) = 3.64, *p* < 0.001
“Rest”-post-pre-Difference	−7.86 ± 9.44	9.4 ± 7.12	*t*(40) = −7.39, *p* < 0.001

### Discussion

This is the first study to investigate difference on the CRAVE-C between athletes and non-athletes. Our results were partially consistent with a previous study indicating that “Move” desire and “Rest” desire after high-intensity interval training significantly decreased and increased, respectively among 9 women (Mean age: 37.9 ± 11.9 years; BMI: 28.2 ± 4.5 kg/m^2^) clinically diagnosed with depression (Filgueiras et al., 2023). Notably, non-athletes and depressed women may not regularly engage in PA, especially moderate-to-vigorous intensity PA, so that HIIT may be too hard to tolerate physiologically. Relatively low tolerance among these unique groups in response to HIIT potentially caused decreased “Move” desire and increased “Rest” desire simultaneously. On the other hand, athletes in the present study demonstrated the opposite effects for both “Move” and “Rest” desires. Such findings may be attributed to the duration of the core training. Specifically, athletes who are typically asked to engage in training sessions of at least 90 min might find it easier to handle than the 30-min HIIT used in the present study, which is consistent with the principle of adaptation (Sandbakk et al., 2013). Results on the between-group difference may be attributed to the fact that athletes have the better ability to recover or rebound from a stressful event like HIIT as compared to non-athletes, which are partially supported by a previous study indicating that Chinese adults who regularly engage in PA seemed to have greater level of exercise tolerance that is positively linked to psychological resilience as the ability to successfully adapt to stress and adversity (Zhang et al., 2022). Collectively, individuals who regularly engage in PA would receive more positive feedback, which potentially contribute to the positive affective exercise experiences so that they are more likely to perceive increased “Move” desire and decreased “Rest” desire after the exercise training.

## Conclusion

The comprehensive research series has broken new ground in examining the intricate relationship between motivational mechanisms and physical activity engagement. Sudy1 translated and validated the CRAVE questionnaire into Chinese (CRAVE-C) to understand PA and SB motivations among Chinese adults. The results showed that CRAVE-C has good psychometric properties, with factor analyses supporting a two-factor model. Cultural differences were noted in interpreting PA intensity. Additionally, the study linked CRAVE-C to affect-based scales, suggesting that positive emotional exercise experiences increase PA motivation. However, the study’s limitations include reliance on self-reporting and a limited demographic sample. Future research should broaden demographic considerations and explore these findings in other populations.

Study 2 examined PA and SB motivations in psychiatric patients, chronic illness patients, and healthy controls using the CRAVE-C questionnaire. It found chronic illness patients had higher PA and SB desires compared to healthy controls, while psychiatric patients exhibited higher SB desire but similar PA desire. This suggests that health awareness and psychological factors significantly influence PA and SB motivations. The study, however, is limited by its modest sample size and narrow condition range, highlighting the need for more expansive future research.

Study 3 identified a negative correlation between cardiorespiratory fitness (CRF) levels (absolute and relative VO₂ max) and post-exercise changes in CRAVE-C “Move” and “Rest” desires. Higher CRF was linked to a greater decrease in “Move” desire and a lesser increase in “Rest” desire post-exercise. Participants with higher CRF showed a slight increase in “Move” desire and a significant decrease in “Rest” desire, suggesting better fitness leads to quicker recovery and less fatigue. However, the study did not examine the duration of these desire changes post-exercise. This gap highlights the need for future research, including comparing professional athletes with non-athletes, to better understand the influence of fitness on post-exercise behavior. These findings offer a foundation for personalized exercise recommendations based on individual fitness levels and recovery patterns.

Study 4 investigated the CRAVE-C scale’s applicability in professional athletes versus healthy non-athletes, focusing on changes in “Move” and “Rest” desires after high-intensity interval exercises. The study involved 22 athletes and 20 university students. Results revealed that athletes experienced a significant increase in “Move” desire and a decrease in “Rest” desire post-training, contrasting with the non-athletes who showed an increase in “Rest” and decrease in “Move” desires. This indicates that high fitness levels in athletes lead to different motivational responses to exercise compared to non-athletes. The study’s findings highlight the CRAVE-C scale’s relevance for professional athletes and suggest the need for further research into different training types to understand exercise motivations better.

### Future research

Moving forward, it is imperative to build upon the foundation laid by this series of studies to further enhance our comprehension of motivational mechanisms. Based on previous findings (Laeremans et al., 2017), future research could benefit from using wearable devices to measure physical activity and sedentary behavior more accurately. Their study highlighted that self-reported moderate-to-vigorous physical activity (MVPA) was significantly lower than the levels measured by wearable devices like Sense Wear, indicating a potential underestimation when relying solely on questionnaires. Furthermore, the poor correlation between self-reported and device-measured sedentary behavior suggests that wearable devices provide a more reliable method for capturing actual activity levels, thereby addressing the limitations and potential biases associated with self-reported measures. Additionally, exploring the desire for physical activity and sedentary behavior through biological approaches, such as imaging techniques like EEG, can offer pivotal insights into the brain’s real-time processing of these desires. Methods like EEG can effectively capture the momentary shifts in cravings measured by the CRAVE scale, thereby expanding the physiological foundation of this assessment tool. This real-time measurement of desire dynamics could further enhance our understanding of how to strategically amplify or suppress an individual’s inclination towards exercise engagement or prolonged sitting. Large-scale studies focusing on individuals with chronic illnesses and/or mental disorders are needed to investigate their desire for physical activity and sedentary behavior, with findings used to customize illness-specific intervention plans. Furthermore, incorporating the CRAVE Scale within exercise prescriptions could assist clinicians and health professionals in designing more nuanced recommendations.

## Data Availability

The datasets presented in this study can be found in online repositories. The names of the repository/repositories and accession number(s) can be found at: DOI 10.17605/OSF.IO/CFPNJ.
