# Supplementary material for: Validation of the CRAVE-C scale in Chinese adults: a four-study examination of competing motivations for physical activity versus rest
Source: Front Psychol. 2024 Oct 23;15:1467949. doi: 10.3389/fpsyg.2024.1467949 (PMC11537887; doi:10.3389/fpsyg.2024.1467949)
Supplement: Supplementary file 1 [file Table_1.docx]

**Appendix**

**Assessing the validity and reliability of the Physical Effort Scale in a Chinese context**

**Methods**

Data for the current study was gathered from 481 participants, as part of Study 1. The measures used in the study encompassed eight items, postulated to reflect two underlying constructs, namely Approach and Avoidance.

The Approach construct was represented by the following items:

"I usually like activities that require physical effort." (V3)

"The idea of exerting physical effort typically appeals to me." (V7)

"I generally enjoy activities that involve physical effort." (V15)

"I am generally willing to participate in activities that involve physical effort." (V18)

Conversely, the Avoid construct was defined by the following items:

"I generally avoid situations that involve physical effort." (V4)

"I tend to stay away from tasks that require physical effort." (V6)

"Exerting physical effort does not appeal to me." (V12)

"I usually dislike activities that involve physical effort." (V17)

The data were subjected to a confirmatory factor analysis (CFA) using the maximum likelihood estimation method. To assess the model fit, we used multiple fit indices, including the chi-square statistic, the Root Mean Square Error of Approximation (RMSEA), the Comparative Fit Index (CFI), the Tucker-Lewis Index (TLI), and the Standardized Root Mean Square Residual (SRMR).

**Results**

***Exploratory Factor Analysis (EFA)***

**Table 1**. Descriptive Statistics and Factor Loadings for the Physical Effort Scale Items (N=481)

tive Statistics and Factor Loadings for the Physical Effort Scale Items (N=481)

| Items | Descriptive Statistics | | | | Factor loading | |
| --- | --- | --- | --- | --- | --- | --- |
|  | Mean | Std | Skew | Kurt | Approach | Avoid |
| 1. I tend to engage in tasks that require physical effort. | 3.39 | 1.09 | -0.3 | -0.54 | -0.108 | 0.722 |
| 2. I generally avoid situations that involve physical effort. | 2.88 | 1.13 | 0.07 | -0.84 | 0.75 | -0.066 |
| 3. I usually like activities that require physical effort. | 3.28 | 1.08 | -0.23 | -0.58 | -0.128 | 0.731 |
| 4. I tend to avoid situations in which I have to exert physical effort. | 2.9 | 1.12 | 0.05 | -0.75 | 0.79 | -0.006 |
| 5. I usually find satisfaction in exerting physical effort. | 3.52 | 1.02 | -0.47 | -0.22 | -0.164 | 0.663 |
| 6. I tend to stay away from tasks that require physical effort. | 2.79 | 1.12 | 0.15 | -0.77 | 0.867 | -0.009 |
| 7. The idea of exerting physical effort usually appeals to me. | 3.28 | 1.04 | -0.19 | -0.54 | -0.15 | 0.831 |
| 8. I tend to avoid tasks that require physical effort. | 2.9 | 1.11 | 0.06 | -0.74 | 0.839 | 0.003 |
| 9. I usually like to engage in physical effort even if there are other possibilities. | 3.18 | 1.07 | -0.17 | -0.55 | 0.061 | 0.908 |
| 10. I generally do not find any satisfaction when I make a physical effort. | 2.56 | 1.05 | 0.33 | -0.43 | 0.835 | 0.142 |
| 11. I tend to search for opportunities to exert physical effort. | 3.1 | 1.01 | -0.06 | -0.39 | 0.075 | 0.894 |
| 12. Exerting physical effort does not appeal to me. | 2.68 | 1.11 | 0.18 | -0.57 | 0.828 | -0.023 |
| 13. I tend to engage in situations in which I have to exert physical effort. | 3.07 | 0.99 | -0.08 | -0.33 | 0.147 | 0.863 |
| 14. When I have to engage in a physical effort, I usually seek to avoid it. | 2.82 | 1.08 | 0.16 | -0.64 | 0.856 | -0.02 |
| 15. I generally enjoy activities that involve physical effort. | 3.31 | 0.98 | -0.51 | -0.05 | -0.078 | 0.786 |
| 16. I usually exert physical effort when there is no other alternative. | 2.96 | 1.07 | -0.08 | -0.65 | 0.691 | -0.06 |
| 17. I usually dislike activities that involve physical effort. | 2.91 | 1.09 | 0.11 | -0.67 | 0.713 | -0.123 |
| 18. I am usually willing to engage in activities that involve physical effort. | 3.28 | 1.02 | -0.26 | -0.45 | -0.062 | 0.761 |

The Kaiser-Meyer-Olkin (KMO) measure of sampling adequacy was calculated and found to be 0.956, exceeding the recommended threshold of .80. Additionally, the Bartlett test returned a significant result [7471.861 (*df =* 153; *p <* 0*.*001)], confirming the adequacy of the correlation matrix. These results suggest that the data were suitable for exploratory factor analysis (EFA). Based on the eigenvalue-greater-than-one rule, the EFA revealed a two-factor structure as the optimal solution. 57.204% of the variance was explained by Factor 1 (eigenvalue = 10.297) and 12.985% of the variance was explained by Factor 2 (eigenvalue = 2.337). As anticipated, the two factors identified exhibited a moderate negative correlation (*r =* -0.64), suggesting potential underlying dynamics between these constructs. The pattern matrix indicated that Factor 1 comprised items 3,7,15,18, while Factor 2 included items 4,6,12,17. All factor loadings for these items were greater than 0.30, supporting their inclusion in their respective factors. In summary, the analysis demonstrated a valid and reliable two-factor solution, accounting for a total of 70.188% of the cumulative variance.

***Confirmatory Factor Analysis (CFA)***

In evaluating the model fit, the results showed a satisfactory alignment with the theoretical expectations. The Comparative Fit Index (CFI) stood at 0.959, closely aligning with the standard threshold, reflecting a sound structural correspondence of the model. Additionally, the Tucker-Lewis Index (TLI) indicated a value of 0.939, further supporting the model’s adequacy. Importantly, the Standardized Root Mean Square Residual (SRMR) was 0.074, well below the recommended upper limit of 0.08. This index underscores the model’s strong fit, with minimal residual variances and covariances. Despite some indices suggesting a less than perfect fit, other indicators show that the model adequately represents the relationships among the observed and latent variables.
